# Identification of novel compounds with prophylactic activity against hypnozoites using a *Plasmodium cynomolgi in vitro* model

**DOI:** 10.1128/aac.01812-24

**Published:** 2025-07-17

**Authors:** Anne-Marie Zeeman, Lars Vermaat, M. Isabel Castellote Alvaro, Nicole M. Van der Werff, Ivonne G. Glas-Nieuwenhuis, Anke Harupa-Chung, Félix Calderón, Fransisco-Javier Gamo, Elena Fernandez Alvaro, Clemens H.M. Kocken

**Affiliations:** 1 Department of Parasitology, Biomedical Primate Research Centre, Rijswijk, The Netherlands; 2 Global Health Medicines R&D, GSK, Madrid, Spain; The Children's Hospital of Philadelphia, Philadelphia, Pennsylvania, USA

**Keywords:** malaria, plasmodium cynomolgi, *in vitro *liver stage assay, hypnozoite, drugs assay, prophylactic, TCAMS, GSK, phenotypic screening, image-based screening

## Abstract

This study aimed to identify new compounds with prophylactic activity against *Plasmodium* liver stages, particularly hypnozoites. A small, focused set of 568 compounds from the GSK open-access TCAMS library was tested *in vitro* against *Plasmodium cynomolgi* liver stages. Among these compounds, 12 showed IC_50_ values below 2 µM against hypnozoites, with the most active compound displaying nanomolar activity. These active compounds could be assigned to/grouped into six chemical clusters. Some compounds could be associated with potential known modes of action such as cytochrome bc1 inhibition or kinase inhibition, whereas the mode of action of other hit compounds is currently unknown. The high hit rate in this screening may be attributed to preselecting compounds based on their activity against gametocytes and liver stages in other *Plasmodium* species. Based on our findings, we suggest that this preselection methodology is appropriate for finding compounds that are active against hypnozoites.

## INTRODUCTION

A safe and highly effective vaccine against malaria could be used to prevent approximately 500,000 deaths and over 200 million malaria cases yearly (1). However, the currently approved vaccine (Mosquirix^T^) (2) works only against *P. falciparum* and has limitations in efficacy and the segment of the population to which it is addressed. Furthermore, it will not help the huge number of people who are already infected, especially people infected with *Plasmodium vivax,* who often suffer from relapsing malaria (3).

The malaria life cycle in mammals is initiated by the bite of an infected mosquito, and sporozoites travel to the liver and develop into schizonts containing tens of thousands of parasites. Parasites exit the liver and enter the bloodstream to infect red blood cells and cause typical malaria symptoms like intermittent high fever, nausea, headache, and in severe cases, organ failure, brain damage, and potentially even death. Relapsing malaria is caused by the reactivation of hypnozoites, dormant liver stage parasites that are described in five *Plasmodium* species (*Plasmodium vivax, (simi)ovale, cynomolgi,* and *fieldi*) (4–8), all of which specifically infect primates, either humans (*vivax, ovale,* and zoonotic *cynomolgi*) or monkeys (*simiovale, cynomolgi,* and *fieldi*). This complicates hypnozoite research as animal models for hypnozoites are restricted to either humanized mice or primates.

Although triggers for reactivation of hypnozoites have been hypothesized, like blood transfusions (9), mosquito bites (10), and exposure to other (bacterial or parasitic) infectious agents (11), including malaria (12), these are not yet confirmed by experimental evidence. The recent publication by Voorberg et al. (13), describing the *in vitro* reactivation of hypnozoites, provides the possibility to investigate triggers for hypnozoite reactivation and develop a “wake and kill” strategy for the elimination of hypnozoites as an alternative to radical cure treatment.

Currently, the standard radical cure treatment is a 7-day (high dose) or 14-day (low dose) regimen with primaquine (14), or a single dose of tafenoquine (15), in combination with the blood-stage drug chloroquine. Both radical cure drugs belong to the class of 8-aminoquinolines, which can cause hemolytic side effects in people with glucose 6-phosphate dehydrogenase deficiency (16). Therefore, there is a need to find alternative drugs that do not carry these risks.

Primaquine and tafenoquine were both discovered utilizing the rhesus/*P. cynomolgi in vivo* model (17). *P. cynomolgi* is a monkey malaria parasite that is closely related to *P. vivax* and is one of the few parasite species that can develop hypnozoites. The benefits and limitations of the *P. cynomolgi* model have been reviewed (18).

To replace ethically undesirable and costly *in vivo* testing in monkeys, we have developed an *in vitro* assay for assessing the activity of compounds against *P. cynomolgi* liver stages (19). This assay can be performed in prophylactic mode, in which test compounds are added shortly after hepatocyte invasion of sporozoites or as a radical cure surrogate with compound treatment from day 5 to day 8 post-infection (20).

Recently, significant progress has been made in the development of high-content screening methods for *P. vivax* liver stages, with the aim of discovering novel compounds for radical cures (21–23). Using this platform, tens of thousands of potential new antimalarials have been screened for their *in vitro* anti-hypnozoite activity. Although a few active compounds have been identified, no clinical candidates have been reported/emerged thus far. The limited availability of patient-derived *P. vivax*-infected blood and cryopreserved human hepatocytes, both essential for *P. vivax* liver stage drug screens, underscores the continued significance of the *P. cynomolgi in vitro* and *in vivo* models. The ability to genetically modify *P. cynomolgi* not only offers the opportunity to enhance drug screening capabilities (24) but also facilitates in-depth studies on hypnozoite biology, as reviewed by Voorberg (25). In addition to the search for new radical cure compounds, there is also a need for new prophylactic drugs to prevent liver infection and thereby prevent relapses caused by hypnozoites. We employed the prophylactic *P. cynomolgi in vitro* liver stage assay to screen a relatively small number of selected compounds for their anti-schizont and -hypnozoite activity and found several active compounds.

Compound preselection was based on antimalarial activity demonstrated in other high-content screening assays that utilized the open-access GSK Tres Cantos Antimalarial set (TCAMS). This library contains 13,533 compounds that inhibit the growth of asexual *P. falciparum* blood stages by at least 80% at 2 µM concentrations (26) and has been used in several other phenotypic antimalarial assays targeting drug-resistant *P. falciparum* asexual blood stages (27), *P. berghei* liver stages (28), and *P. falciparum* gametocytes (29, 30).

## MATERIALS AND METHODS

### Experimental set-up

In search of new compounds to replace the 8-aminoquinolines primaquine and tafenoquine *P. vivax* prophylactics, the focused library of 568 compounds was screened in the *P. cynomolgi in vitro* liver stage assay, with particular interest in compounds exerting prophylactic anti-hypnozoite activity. The imaging-based primary screening was performed in a 96-well plate format at 10 µM compound concentration in duplicate, with the PI4K inhibitor MMV390048 (1 µM) as positive control. The two licensed anti-hypnozoite compounds primaquine and tafenoquine need to be metabolized to exert antimalarial activity, whereas the GSK compounds tested in this setting most likely have a direct effect on the liver-stage parasite. We therefore used the PI4K inhibitor MMV390048, previously identified as a strong anti-hypnozoite active compound, as a positive control. Primary rhesus monkey hepatocytes were infected with *P. cynomolgi* sporozoites and treated with a compound from 1.5 h post-infection (pi) until day 6 pi when cells were fixed and stained with anti-*P*. *cynomolgi* Hsp70.1 antibodies to visualize the parasites. Images were acquired on the Operetta high-content imager (Perkin-Elmer) and analyzed using Harmony software (Perkin-Elmer) to determine the number of liver stages per well, subdivided into small (hypnozoites) and large (developing schizonts) forms (19). Compounds that led to a reduction of either the total number of liver stages or small forms by at least 50% compared with the untreated control were selected as primary hits. Hits and analogs within the same chemical cluster were retested at 3 concentrations (10, 1, and 0.1 µM) in duplicate and were progressed to full dose-response assays (in triplicates) if they eliminated more than 75% of the liver stages.

### Animal use

Nonhuman primates were used because no other models (*in vitro* or *in vivo*) were suitable for the aims of this project. The research protocol was approved by the central committee for animal experiments (CCD license number AVD5020020172664), and the subprotocol was approved by the local independent ethical committee constituted conform Dutch law (BPRC Dier Experimenten Commissie, DEC; agreement number #708 and #007C) prior to the start of the experiments. All experiments were performed according to Dutch and European laws. The Council of the Association for Assessment and Accreditation of Laboratory Animal Care (AAALAC International) has awarded BPRC full accreditation. Thus, BPRC is fully compliant with the international demands on animal studies and welfare as set forth by the European Council Directive 2010/63/EU and Convention ETS 123, including the revised Supplemental data as well as the “Standard for Humane Care and Use of Laboratory Animals by Foreign Institutions” identification number A5539-01, provided by the Department of Health and Human Services of the United States of America’s National Institutes of Health (NIH) and Dutch implementing legislation. Only healthy animals were included in the experiments. The rhesus monkeys used in this study (Macaca mulatta, *n* = 7, either gender, age 4–16 years, Indian origin) were captive-bred and socially housed. Animal housing was according to the international guidelines for nonhuman primate care and use. Besides their standard feeding regime and drinking water *ad libitum* via an automatic watering system, the animals followed an environmental enrichment program. Monkeys were trained to voluntarily present for thigh pricks and were rewarded afterward. All intravenous injections and large blood collections were performed under ketamine sedation, and all efforts were made to minimize suffering. Liver cells were derived from in-house frozen batches of hepatocytes or from freshly collected liver lobes from monkeys that were euthanized in the course of unrelated studies (ethically approved by the BPRC DEC) or euthanized for medical reasons, as assessed by a veterinarian. Therefore, none of the animals from which liver lobes were derived were specifically used for this work, according to the 3R rule, thereby reducing the number of animals used. Euthanasia was performed under ketamine sedation (10 mg/kg) and was induced by intracardiac injection of Euthasol 20%, containing pentobarbital.

GSK is committed to the replacement, reduction, and refinement of animal studies (3Rs). Non-animal models and alternative technologies are part of our strategy and are employed where possible. When animals are required, the application of robust study design principles and peer review minimizes animal use, reduces harm, and improves benefit in studies.

All animal studies were ethically reviewed and carried out in accordance with European Directive 2010/63/EU and the GSK Policy on the Care, Welfare and Treatment of Animals.

### *P. cynomolgi* sporozoite production

Blood-stage infections were initiated in rhesus monkeys (Macaca mulatta) by intravenous injection of 1 × 10^6^ *P. cynomolgi* M strain parasites from a cryopreserved stock. Parasitemia was monitored by Giemsa-stained smears prepared from a droplet of blood. Around peak parasitemia, monkeys were bled on 2 consecutive days, generally at days 11 and 12 post-infection, to feed mosquitoes. Monkeys were cured of Plasmodium blood-stage infection by intramuscular treatment with chloroquine (7.5 mg/kg) on 3 consecutive days. Bleeds and infection of the monkey with blood stage parasites were performed under ketamine sedation; thigh pricks and intramuscular injections were performed on alert animals, which were trained to undergo these procedures by positive reinforcement. Per blood sample, approximately 600 mosquitoes (2–5 days old female *Anopheles stephensi* mosquitoes, Sind-Kasur strain Nijmegen. Nijmegen UMC St. Radboud, Department of Medical Microbiology) were fed using a glass feeder system (31). Mosquitoes were kept under standard conditions (25°C, 80% humidity) and were given cotton wool with 5% glucose in tap water daily. Approximately 1 week after feeding on the infected blood, midgut oocysts were counted in ten mosquitoes, and the remaining mosquitoes were given an uninfected blood meal (usually with reconstituted blood, consisting of 50% rhesus red blood cells and 50% human serum (A+)) to synchronize sporozoite invasion of the salivary glands (31).

### Primary hepatocytes

Livers were collected from monkeys that were euthanized for unrelated studies (which were ethically approved by the Central Committee for Animal Testing [CCD]) or euthanized for medical reasons, as assessed by a veterinarian. Primary hepatocytes from *Macaca mulatta* were isolated freshly as described before (32) and resuspended in William’s B medium (William’s E with Glutamax containing 10% human serum (A+), 1% MEM non-essential amino acids, 2% penicillin/streptomycin, 1% insulin/transferrin/selenium, 1% sodium pyruvate, 50 µM β-mercapto-ethanol, and 0.05 µM hydrocortisone). Hepatocytes were seeded in collagen-coated 96-well plates (Perkin Elmer Cell Carrier plates) at a concentration of approximately 70 × 10^6^ cells/well. Following attachment, the medium was replaced by William’s B containing 2% dimethylsulfoxide (DMSO) to prevent hepatocyte dedifferentiation. The cells were used for *in vitro* infection with sporozoites within 4 days after isolation.

### Compound selection and construction of the library

To discover new starting points to develop anti-hypnozoite drugs, we assembled a set of 568 compounds with antimalarial activity in *P. falciparum* asexual stages. The set included chemical diversity selected following different criteria: (i) compounds from the Tres Cantos Antimalarial Set active against *P. falciparum* gametocytes, (ii) compounds from the TCAMS active against *Plasmodium yoelii* hepatic forms, and (iii) compounds from GSK extended collection active in *P. falciparum* asexual blood stages, which represented interesting and novel chemical diversity with an unknown mode of action.

### Sporozoite isolation, hepatocyte infection, and drug treatment

Two weeks after the mosquitoes ingested blood infected with *P. cynomolgi* blood-stage parasites, salivary gland sporozoites were isolated and used for hepatocyte infection (19). Prior to sporozoite inoculation, hepatocytes were washed in William’s B medium, followed by sporozoite inoculation at ±50 × 10^3^ sporozoites per well in 96-well plates. Plates were spun at RT at 500 × *g* for 10 min and placed in a humidified 37°C incubator at 5% CO_2_ for 2 h to allow for sporozoite invasion, after which the medium was refreshed. Compounds were diluted in William’s B medium to the desired final concentration and added to the culture at the first medium refreshment. Compound dilutions were stored at 4°C between refreshments. Each assay plate contained the following controls: uninfected hepatocytes, infected untreated wells, and infected wells treated with 1 µM of MMV390048, a PI4K inhibitor, as growth inhibition control (33). Regular medium changes were performed until day 6 post-infection (pi) when plates were fixed with 4% paraformaldehyde and stained with an antibody directed against *P. cynomolgi* Hsp70.1 (see below).

### Immunofluorescent detection of intracellular parasites

Intracellular *P. cynomolgi* malaria parasites were stained as described previously (19) using 1:10,000 diluted rabbit polyclonal anti-*P*. *cynomolgi* Hsp70.1 antibody (34) and Alexa568-labeled Goat-anti-rabbit IgG secondary antibody (1:1,000) in dilution buffer (1% BSA and 0.3% Triton X-100 in PBS). Nuclei were stained with 2 µM DAPI (4′,6-diamidino-2-phenylindole). The number of intracellular parasites was determined using a high-content imaging system (Operetta, Perkin-Elmer) (19), and images were analyzed as previously described (19) using Harmony software (Perkin-Elmer), defining hypnozoites as exo-erythrocytic forms (EEF) smaller than 40 µm^2^.

## RESULTS

### Library selection and construction

The GSK TCAMS library comprises 13,533 compounds selected as a result of a whole cell screening against *Plasmodium* blood stages (26). To build a smaller set and increase the probability of identifying compounds with activity against hypnozoites, we selected compounds with confirmed phenotypic activity in other antimalarial assays. This first selection criterion ensures that the compounds meet the minimum requirement for both solubility and permeability for activity in these types of assays. In terms of anti-parasitological profile, we prioritized compounds with demonstrated activity against liver stages (ensuring their ability to target parasites residing inside hepatocytes) and/or mature stage V gametocytes, which present a low metabolic activity, like hypnozoites. To expand the chemical diversity and explore a broader range of compounds, the list was topped/enriched with analogs of the phenotypic hits and additional compounds from the collection. Information from published screens and results from GSK’s internal lead optimization programs targeting liver stages and/or gametocytes were utilized to assess compound availability within the global GSK compound collection. As a result, a library of 568 compounds was compiled, consisting of 285 chemical clusters. Among these clusters, 43 were considered series with two or more representatives, whereas the remaining 242 were classified as singletons.

### Results of the single concentration testing (screening)

In the single-dose (10 µM) prophylactic *in vitro* liver stage screening of 568 compounds, we found that 48 compounds inhibited either the total number of liver stages (*n* = 47 compounds) or hypnozoites only (*n* = 1 compound) by more than 50% compared with the untreated control (see Supplemental data for an overview of the compound test results: primary hits, 10-fold dilutions, and dose-response curves). This primary hit rate of 8.3% is surprisingly high for anti-hypnozoite drug screening. In reported *P. vivax* prophylactic liver stage drug screens (21, 23), confirmed active compounds were limited to ionophores, PI4K inhibitors, and 8-aminoquinolines. Similarly, in our own *P. cynomolgi* prophylactic and radical cure screens, where we evaluated over 12,000 compounds (manuscript in preparation), we also found that only PI4K-inhibitors and 8-aminoquinolines were prophylactically active against hypnozoites. If high *in vitro* metabolic stability in liver microsomes is included in the selection of compounds (data available for 163 out of the 568 screened), the primary hit rate increases by 15% (25/163). In fact, for a confirmed active chemotype (cluster 1, Table 1), a subset of 49 analogs within the same range of potencies in erythrocytic stages (*Pf*IC_50_ 0.01–0.1 µM) and low degradation in human microsomes resulted in a rate of more than 50% (26/49) in the primary screening. The 48 primary hit compounds were chemically classified into 19 clusters (Table 1), with the objective to progress, at least one hit per cluster progressed to the 10-fold-dilution retest (Table 1, column B). In low-populated clusters and singletons, analogs from the GSK collection were included when available (Table 1, column C). In addition, cluster 7 was discarded (as only one compound out of 39 analogs evaluated in the primary screening showed activity), and cluster 11 did not further progress due to compound availability issues. Hit confirmation was performed in three 10-fold dilution series of 10, 1, and 0.1 µM in prophylactic mode. For the primary hit confirmation, a cutoff of 75% inhibition at 10 µM was chosen to select the more potent compounds.

**TABLE 1:** Compound progression from primary screening into 3-dilution test

Cluster number (Chemical class/potential mode of action)	A Active compounds (Primary hits/number of compounds screened)	B Active compounds in three 10-fold dil. screen/number of compounds evaluated	C Active analogs/number of compounds evaluated
Cluster 1 (Aminoindole/unknown)	26/59	4/5	0
Cluster 2 (Thiazole/kinase scaffold)	2/2	2/2	2/2
Cluster 3 (Quinolone/bc1)	2/3	1/2	1/1
Cluster 4 (2,4-disubstituted 7-Azaindoles/kinase scaffold)	1/5	1/1	1/1
Cluster 5 (3,5-disubstituted 7-Azaindoles/kinase scaffold)	1/4	1/1	0
Cluster 6 (Naphthyridine /kinase scaffold)	1/1	1/1	0
Cluster 7 (Thiotriazole/ATP4)	1/39	0	0
Cluster 8 (Aminoquinoxalines/kinase scaffold)	1/1	1/1	0/2
Cluster 9 (Benzimidazole/unknown)	1/1	0/1	0/3
Cluster 10 (Aminoxadiazole/unknown)	2/2	1/2	0/1
Cluster 11 (4,6-Diaminopyrimidine/unknown)	1/1	0	0
Cluster 12 (Pyridopyrimidinone/kinase scaffold)	1/2	0/1	1/3
Cluster 13 (Thiopyrimidines/unknown)	1/2	0/1	0
Cluster 14 (Heteroaryl benzamide/unknown)	1/1	0/1	0/1
Cluster 15 (Furan- carboxamide/ unknown)	1/1	1/1	1/1
Cluster 16 (Aminoacetamide/unknown)	1/4	0/1	0
Cluster 17 (Aryl thiazole/unknown)	1/1	0/1	1/2
Cluster 18 (Tricyclic system /unknown)	1/1	0/1	0
Cluster 19 (Aryl urea/unknown)	1/1	0/1	0/1

### Hit confirmation using dose-response curves

Forty-two compounds (24 primary hits representing 17 clusters + 18 analogs from 11 of those clusters, Table 1 columns B and C) were retested in three dilutions of 10, 1, and 0.1 µM.

Of the 42 compounds that progressed to 3-concentration hit confirmation, 21 (including the blinded positive control MMV390048 [33]) were confirmed to kill at least 75% of the total number of liver stages in one or more of the three concentrations tested. For the 24 primary hits, activity was confirmed for 13 compounds, using the higher cutoff level. Seven out of 18 hit analogs eliminated >75% of the liver stage parasites in the three 10-fold dilution series.

**Fig 1:**
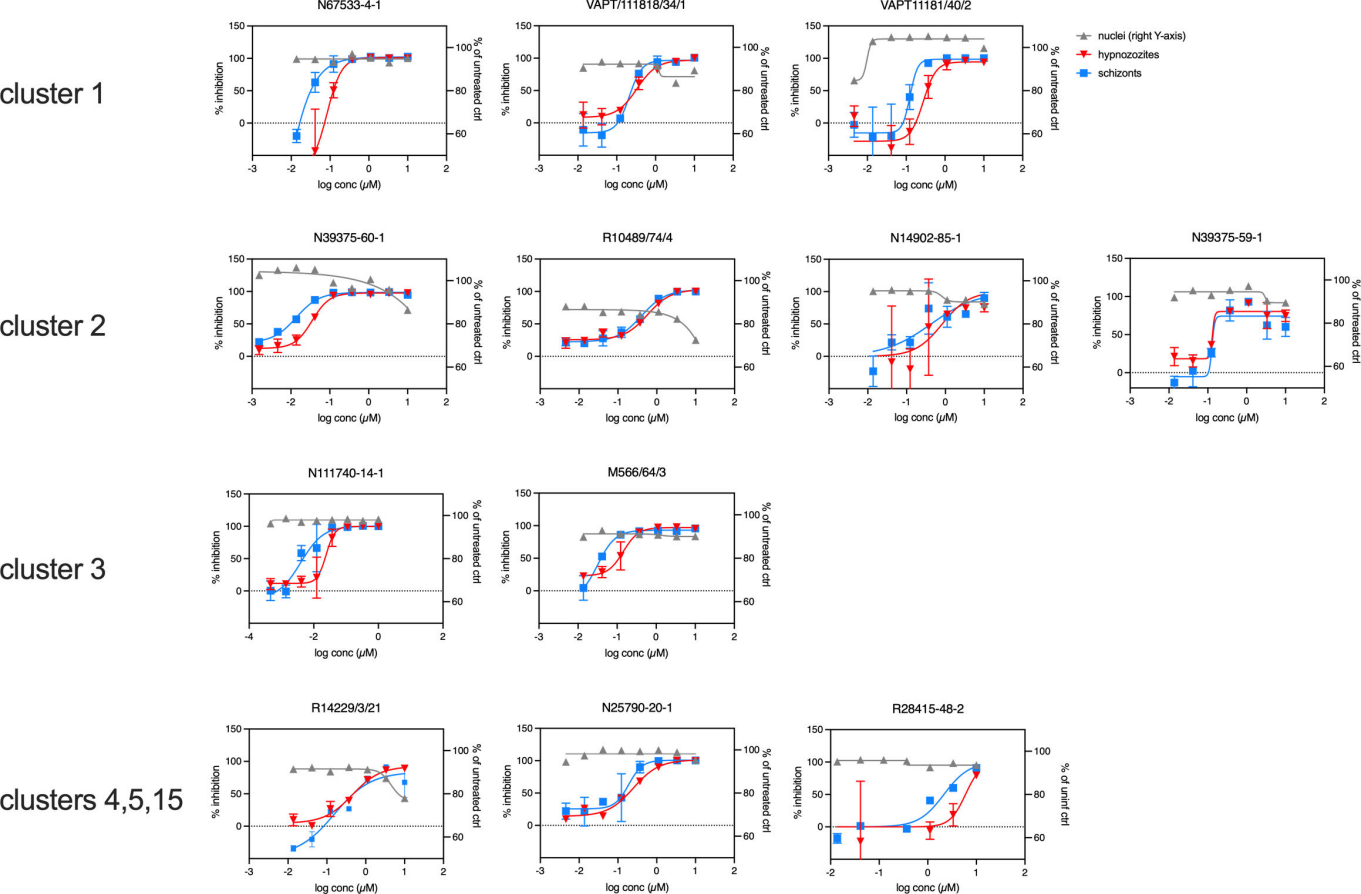
Individual dose-response curves of 12 compounds (arranged by compound cluster) with sub-micromolar activity against hypnozoites in the prophylactic (drug treatment from d0-6pi) *in vitro P. cynomolgi* liver stage assay. Parasites were fixed on d6 pi, and stained with anti-*P*. *cynomolgi* Hsp70 antibodies and Alexa568-labeled secondary antibodies and analyzed using a high-content screening microscope (Operetta, Perkin Elmer). Curves represent the mean ± SD of triplicate measurements. Activity against *P. cynomolgi* (left Y-axis, %inhibition) liver schizonts (blue lines, squares) and hypnozoites (red lines, triangles) was determined. Dose-dependent reduction of the nuclei counts (right Y-axis) hints toward the cytotoxicity of the compound (evident in R10489/74/4).

**Fig 2:**
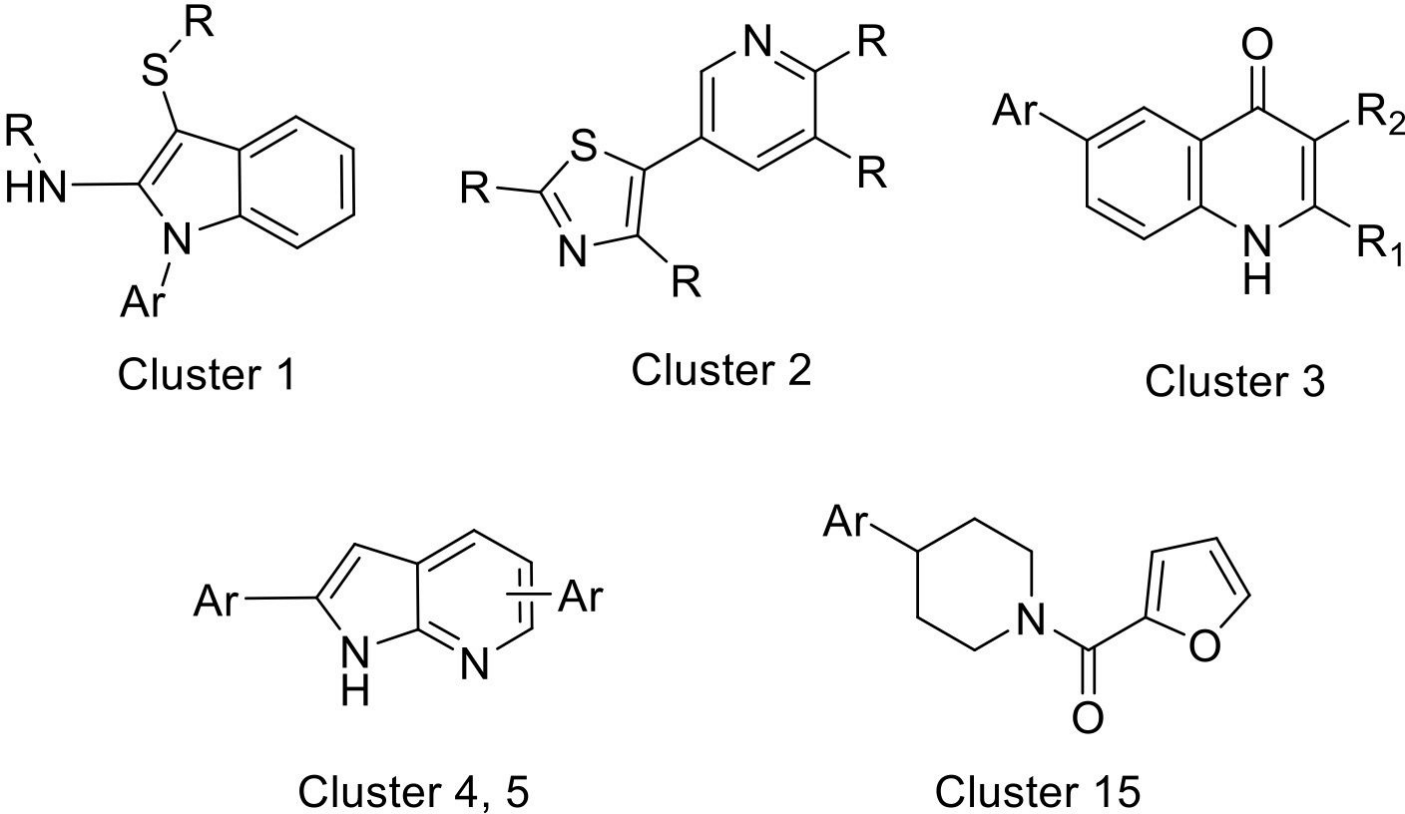
Confirmed chemotypes with *P. cynomolgi* liver stage activity. Cluster 1: aminoindoles; cluster 2: thiazoles; cluster 3: quinolones; cluster 4/5: 7-aza-indoles; and cluster 15: furan-carboxamide. Structures of selected compounds are available in the supplementary data (Fig. S1).

Twenty of the 21 confirmed compounds (excluding the blinded positive control MMV390048) progressed to dose-response evaluation. On every plate of the dose-response evaluation, we included the positive control MMV390048; this compound had a highly reproducible IC_50_ of 42.6 ± 15.3 nM for hypnozoites and 24.9 ± 9.7 nM for developing EEF. IC_50_s were calculated using curve fitting (non-linear fit of [inhibitor] vs. response, four-parameter fit). Twelve of the newly identified compounds were identified with IC_50_ values for liver stages below 2 µM (Table 2, Fig. 1). These compounds belonged to six different chemical families (Fig. 2), three of them being singletons (Table 2). Preliminary *in vitro* toxicity data available showed a window of at least one order of magnitude compared with their hypnozoite activity. List of hits in the first screening, analogues selected for the 3-dilutions series and IC50 tests are provided in Supplemental data.

**TABLE 2:** Prophylactic IC_50_ values of the 12 most active (IC_50_ <2 µM) compounds in the dose-response test^*a*^

Compound name	Cluster	Hypnozoite		Schizont		Maximum efficacy (%inhib)	
		IC_50_ (µM)	Fit (R squared)	IC_50_ (µM)	Fit (R squared)	Hypnozoite	Schizont
N67533-4-1	1	0.083	0.9998	0.0053	0.9997	100	100
VAPT/111818/34/1	1	0.33	0.9915	0.21	0.9958	100	100
VAPT/111818/40/2	1	0.28	0.9097	0.13	0.9879	94	100
N14902-85-1	2	0.066	0.9332	~ 1.3e-010	0.9028	75	90
N39375-59-1	2	~ 0.13	0.9663	~ 0.13	0.9099	75	60
N39375-60-1^*b*^	2	0.035	0.9974	0.014	0.9934	98	95
R10489/74/4^*b*^	2	0.58	0.9847	0.41	0.9961	100	100
M566/64/3	3	0.14	0.9987	0.032	0.9985	92	96
N11740-14-1	3	0.024	0.9994	0.0040	0.9608	100	100
R14229/3/21^*b*^	4	0.45	0.9803	0.16	0.9376	89	68
N25790-20-1	5	0.25	0.9811	0.19	0.9869	100	100
N28415-48-2	15	1.9	0.7145	0.79	0.7134	79	91

^*a*^ Compounds were tested in triplicate.

^*b*^ Slight cytotoxicity (reduction of the number of hepatocyte nuclei) observed at the highest concentration.

## DISCUSSION

Using *P. cynomolgi in vitro* liver stage cultures, we successfully screened a small, focused library of 568 compounds for prophylactic activity against hypnozoites and schizonts. In this small-scale screen in prophylactic mode (treating from 1.5 h post-infection to day 6 pi), with compounds tested in duplicate at 10 µM single concentration, 48 hits (defined as killing over 50% of the hypnozoite population) were identified. Likely, the criteria for library selection (active in *P. falciparum* blood stage and/or gametocyte assays and/or active in *P. berghei* liver stage assay) led to an enrichment in compounds active against *P. cynomolgi* hepatic forms, including hypnozoites. By far, the highest number of hit compounds was derived from one cluster, the promising new antimalarial class of amino-indole compounds (35) (59 compounds tested and 26 hits). To reduce the redundancy within this cluster, we selected only five amino indole compounds for the follow-up experiments. From the other chemical clusters, up to four compounds were selected for hit confirmation in three 10-fold dilutions of 10, 1, and 0.1 µM. Due to the lack of availability of some compounds, hit confirmation was performed with 24 compounds identical to the primary hit, plus 18 closely related analogs.

Of the 42 compounds tested in the 10-fold dilution series, 21 (including the blinded control MMV390048) were confirmed as hits using the more restrictive criteria (75% reduction of the hypnozoites compared with untreated control). From the compounds that were identical between screening and hit confirmation, 14 of the 24 (58%) were confirmed as hits in the 10-fold dilutions test. This is not surprising, as for the primary screening, the stringency was lower in comparison to the hit confirmation. Six of the 18 (33%) analogs tested positive in the 10-fold dilutions test.

Most of the potential hits that were not confirmed in the 3-dilutions test were compounds that were not very active in the primary screening or compounds that showed large variations between the duplicate measurements.

Dose-response testing of the 20 confirmed hit compounds showed very strong anti-hypnozoite activity of 12 compounds, with IC_50_s below 2 µM against hypnozoites. The compounds were derived from five different chemical series (6 clusters). According to previous reports, these chemical classes might be acting through different mechanisms of action, a hypothesis that needs to be confirmed. For example, thiazole compounds bind to the DHFR enzyme (36), whereas quinolones are inhibitors of the plasmodial electron transport chain, targeting the parasite’s *bc*1 complex (37). It is interesting to note that *bc*1 complex inhibitors (cluster 3) show prophylactic activity against hypnozoites, whereas a previous report on antimalarial activity of *bc*1 inhibitors only demonstrated prophylactic activity on developing liver stage schizonts, not on hypnozoites (38). This suggests that the hypnozoite *bc*1 complex could be a potential target for anti-hypnozoite drug development. Azaindoles are known as inhibitors of CAMKK2 serine/threonine kinase (39). Amino-indoles have multiple modes of action, like inhibition of hemozoin formation and PfATP4 inhibition (40), but the exact target of this compound series is not yet identified. Also, we need to keep in mind that the compounds could also target the host cell and kill the parasite via an indirect (host-derived) pathway. Thus, more studies on the mode of activity of the active compounds are needed to elucidate new molecular targets for killing hypnozoites.

The identification of 12 very active compounds from a small (only 568 compounds) library was quite surprising. Likely, the compound preselection strategy based on selecting compounds with appropriate properties to present activity in a phenotypic screening and bias towards gametocytocidal activity and liver stage activity in other Plasmodium species resulted in enrichment for compounds with anti-hypnozoite activity.

Of the 20 compounds selected for dose-response testing, one compound (SO108632-083A1) was not active at all in this assay, whereas in the three dilutions experiment, activity was detected only at the highest concentration. Also, compounds FMAJ/280/67/1 and ST/1413689 were not very active in the dose-response test. These compounds, like SO108632-083A1, only showed a reduction in the number of liver-stage parasites at the highest concentration in the three dilutions test. The discrepancy in activity could be due to differences in hepatocyte batches between the different experiments, resulting in borderline detectable activity in one experiment and low to no activity of the compound in another experiment.

Up to now, we have only found 8-aminoquinolines, PI4K inhibitors, and Monensin A (and other ionophores) to act against hypnozoites in prophylactic *P. cynomolgi* assays. Therefore, it is noteworthy to find such a high number of chemically distinct compounds in our current study that showed prophylactic activity in the *P. cynomolgi* liver stage assay.

Subsequent lead optimization may result in improved activity, especially if compound targets are identified. *In vitro*/*in silico* compound optimization should then precede the *in vitro* hypnozoite assay testing.

## SUPPLEMENTAL MATERIAL

Fig. S1 aac.01812-24-s0001.docx Structure formulas of selected compounds that were identified as prophylactically active anti-hypnozoite drugs.

Supplemental data aac.01812-24-s0002.xls Individual compounds result in the screening, linking results from primary screen to hit confirmation and IC50.

ASM does not own the copyrights to Supplemental Material that may be linked to, or accessed through, an article. The authors have granted ASM a non-exclusive, world-wide license to publish the Supplemental Material files. Please contact the corresponding author directly for reuse.
