# Supplementary material for: Identification of novel compounds with prophylactic activity against hypnozoites using a Plasmodium cynomolgi in vitro model
Source: Antimicrob Agents Chemother. 2025 Jul 17;69(8):e01812-24. doi: 10.1128/aac.01812-24 (PMC12327006; doi:10.1128/aac.01812-24)
Supplement: Fig. S1 — Structure formulas of selected compounds that were identified as prophylactically active anti-hypnozoite drugs. [file aac.01812-24-s0001.docx]

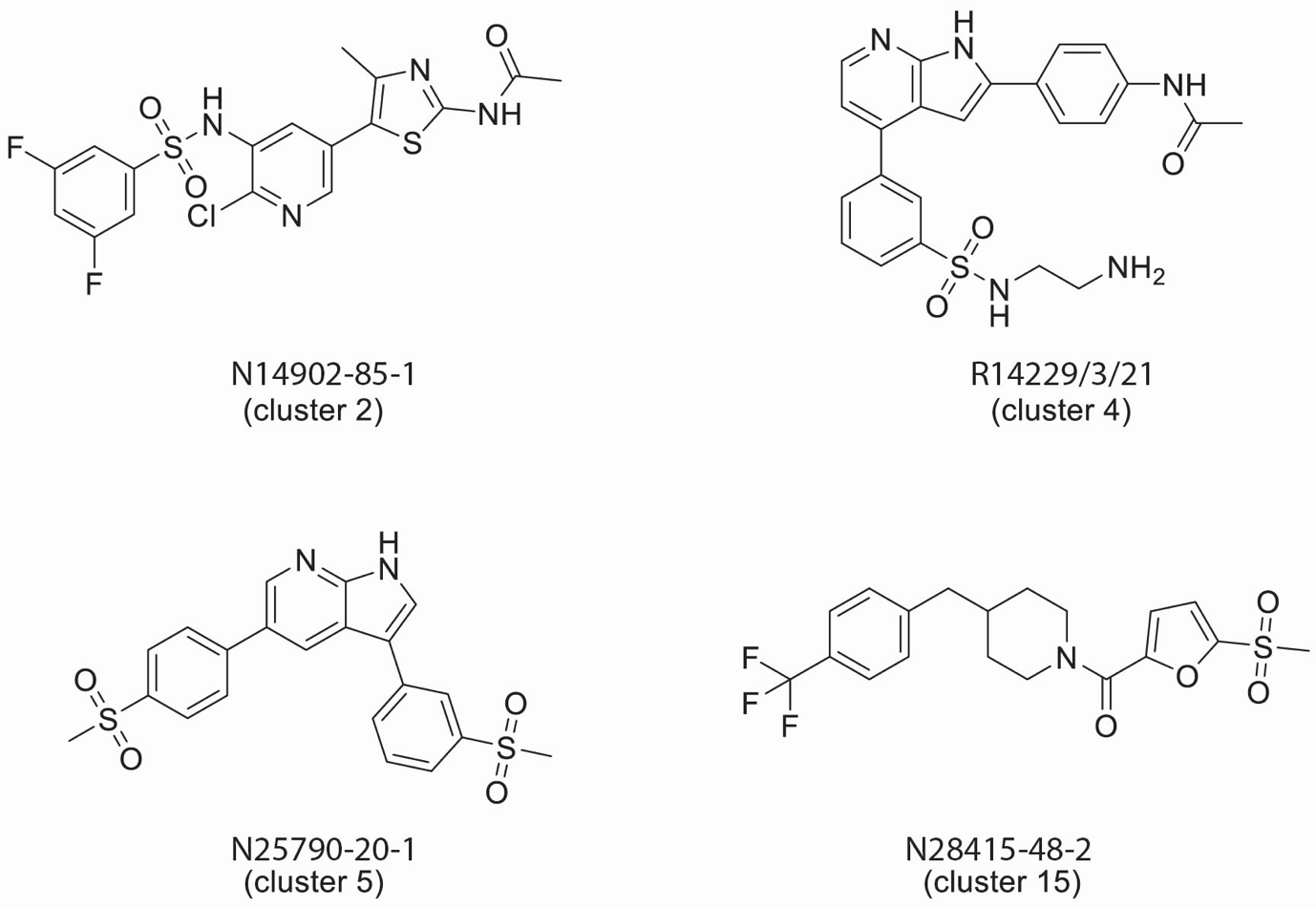


Figure 1S: Structure formulas of selected compounds that were identified as prophylactically active anti-hypnozoite drugs. Cluster 2: Thiazole/Kinase compounds, cluster 4: 2,4-disubstituted 7-Azaindoles/kinase scaffold, cluster 5: 3,5-disubstituted 7-Azaindoles/kinase scaffold and cluster 15: Furan- carboxamide.
